# Development of a new outcome prediction model for Chinese patients with penile squamous cell carcinoma based on preoperative serum C-reactive protein, body mass index, and standard pathological risk factors: the TNCB score group system

**DOI:** 10.18632/oncotarget.8037

**Published:** 2016-03-11

**Authors:** Zai-Shang Li, Peng Chen, Kai Yao, Bin Wang, Jing Li, Qi-Wu Mi, Xiao-Feng Chen, Qi Zhao, Yong-Hong Li, Jie-Ping Chen, Chuang-Zhong Deng, Yun-Lin Ye, Ming-Zhu Zhong, Zhuo-Wei Liu, Zi-Ke Qin, Xiang-Tian Lin, Wei-Cong Liang, Hui Han, Fang-Jian Zhou

**Affiliations:** ^1^ Department of Urology, Sun Yat-sen University Cancer Center, Guangzhou, P. R. China; ^2^ State Key Laboratory of Oncology in Southern China, Guangzhou, P. R. China; ^3^ Collaborative Innovation Center of Cancer Medicine, Guangzhou, P. R. China; ^4^ Department of Urology, Affiliated Tumor Hospital of Xinjiang Medical University, Urumchi, P. R. China; ^5^ Department of Urology, Cancer Center of Guangzhou Medical University, Guangzhou, P. R. China; ^6^ Department of Urology, Dong Guan People's Hospital, Guang Dong, P. R. China; ^7^ Department of Urology, The First People's Hospital of Chenzhou, Chenzhou, P. R. China; ^8^ School of Life Science, Sun Yat-sen University, School of Life Science, Guang Dong, P. R. China; ^9^ Department of Urology, The People's Hospital of Jiangmen, Jiangmen, P. R. China; ^10^ Zhongshan School of Medicine, Sun Yat-sen University, Guang Dong, P. R. China

**Keywords:** penile neoplasms, neoplasm staging, prognosis, body mass index, C-reactive protein

## Abstract

**Purpose:**

To determine the predictive value and feasibility of the new outcome prediction model for Chinese patients with penile squamous cell carcinoma.

**Results:**

The 3-year disease-specific survival (DSS) was 92.3% in patients with < 8.70 mg/L CRP and 54.9% in those with elevated CRP (*P* < 0.001). The 3-year DSS was 86.5% in patients with a BMI < 22.6 Kg/m2 and 69.9% in those with a higher BMI (*P* = 0.025). In a multivariate analysis, pathological T stage (*P* < 0.001), pathological N stage (*P* = 0.002), BMI (*P* = 0.002), and CRP (*P* = 0.004) were independent predictors of DSS. A new scoring model was developed, consisting of BMI, CRP, and tumor T and N classification. In our study, we found that the addition of the above-mentioned parameters significantly increased the predictive accuracy of the system of the American Joint Committee on Cancer (AJCC) anatomic stage group. The accuracy of the new prediction category was verified.

**Methods:**

A total of 172 Chinese patients with penile squamous cell cancer were analyzed retrospectively between November 2005 and November 2014. Statistical data analysis was conducted using the nonparametric method. Survival analysis was performed with the log-rank test and the Cox proportional hazard model. Based on regression estimates of significant parameters in multivariate analysis, a new BMI-, CRP- and pathologic factors-based scoring model was developed to predict disease-specific outcomes. The predictive accuracy of the model was evaluated using the internal and external validation.

**Conclusion:**

The present study demonstrated that the TNCB score group system maybe a precise and easy to use tool for predicting outcomes in Chinese penile squamous cell carcinoma patients.

## INTRODUCTION

More than 95% of penile cancers are squamous cell carcinomas (SCC) [1, 2]. It is well known that lymph node metastasis is the most important variable affecting survival [3-5]. Almost all studies in this area investigated survival outcomes in relation to lymph node- or other nodal-related prognostic factors [1-6], and several prediction models have recently been published to predict the outcome of penile SCC cases based on these prognostic factors [7-9]. However, classical molecular markers and physical status factors are not clinically useful for the prognosis in SCC of the penis.

Serum C-reactive protein (CRP) is an indicator of acute or chronic inflammation [10]. Elevated CRP concentrations have been correlated independently with tumor load and disease progression, which have also been shown to correlate with survival in various cancers [11, 12]. Recently, two studies showed that the CRP level correlates with tumor burden in cases of penile cancer [13, 14].In addition, weight has been thought to be an independent risk factor in patients with cancer [15]. Beyond associations between weight and cancer incidence, body mass index (BMI) is also associated with worse prognoses and outcomes for patients with cancer [16-18]. However, one study reported a negative correlation between BMI and disease-specific survival (DSS) in patients with penile cancer [19].

To our knowledge, no studies have simultaneously measured both molecular markers and physical status in penile SCC patients whose clinical course may frequently be complicated by infection. We analyzed the correlation between preoperative serum CRP and clinic-pathological parameters in penile SCC patients and aimed to determine its clinical significance as a potential predictor for poor prognostic outcome.

## RESULTS

### Patient characteristics

Of the 172 patients, 19 (11.6%) patients had local excision with circumcision, 116 (67.4%) patients had partial penile amputation, and 37 (21.5%) patients had total penile amputation. The 143 (83.1%) eligible patients underwent bilateral ILND, and 46 (26.7%) patients underwent pelvic lymphadenectomy. The median numbers of positive and removed inguinal LNs were 2 (IQR: 1–13) and 27 (IQR: 5–55), respectively. Adjuvant therapy was performed in 45 (26.2%) patients. The potential level of preoperative CRP was 8.70 mg/L. During the follow-up, 28 patients died of penile cancer at a mean of 21.5 months (median 12.9 months, range 2.2 month to 116.9 months). The clinic-pathological characteristics of the patients are presented in Table 1.

**Table 1:** Clinic-pathological characteristics and univariate log-rank test of prognostic covariates in 172 patients

Variable	N (%)	1-year DSS (95% CI)	3-year DSS (95% CI)	*P*-value
Age at surgery, year, median (range)	52 (25-86)			0.365
≥ 52	87 (50.6)	82.0 (73.7-90.2)	77.9 (68.3-87.5)	
< 52	85 (49.4)	89.2 (82.1-96.3)	75.7 (59.4-91.9)	
BMI, kg/m2, median (range)	22.8 (13.6-36.7)			0.025
≥ 22.6	87 (50.6)	90.9 (84.4-97.4)	86.5 (77.9-95.1)	
< 22.6	85 (49.4)	79.9 (71.1-88.7)	69.9 (56.6-83.2)	
Smoking History				0.472
Yes	103 (59.9)	82.9 (75.2-90.5)	78.2 (68.4-88.0)	
No	69 (40.1)	89.2 (81.6-96.8)	78.6 (65.1-92.1)	
Phimosis				0.606
Yes	127 (73.8)	84.6 (77.9-91.2)	81.3 (73.5-89.1)	
No	45 (26.2)	88.6 (79.2-98.0)	66.4 (41.7-91.1)	
Pelvic LNM (n)*				0.247
Yes	18 (10.5)	77.8 (58.6-97.0)	77.8 (58.6-97.0)	
No	154 (89.5)	86.6 (80.9-92.3)	78.8 (70.4-87.2)	
T (n)				<0.001
≤ T1	57 (33.1)	98.2 (94.8-100.0)	98.2 (94.8-100.0)	
T2	90 (52.3)	86.0 (78.2-93.8)	77.1 (65.1-89.1)	
≥ T3	25 (14.5)	53.8 (33.6-74.0)	35.8 (11.5-60.1)	
G (n)				0.006
G1	102 (59.3)	90.3 (84.2-96.4)	84.1 (73.7-94.5)	
G2	55 (31.9)	85.0 (75.4-94.6)	74.7(60.8-88.6)	
G3	15 (8.7)	56.2 (29.5-82.9)	56.2 (29.5-82.9)	
N (n)*				<0.001
N0	88(51.2)	98.9 (96.7-100.0)	98.9 (96.7-100.0)	
N+	84 (48.8)	71.8 (61.6-82.0)	57.3 (43.0-71.6)	
CRP (n)				<0.001
Positive (≥ 8.70 mg/L)	59 (34.3)	69.8 (57.6-82.0)	54.9 (38.4-71.4)	
Negative (< 8.70 mg/L)	113 (65.7)	93.9 (89.2-98.6)	92.3 (86.6-98.0)	

N*, N+ including pN1, pN2, pN3.

**Figure 1:**
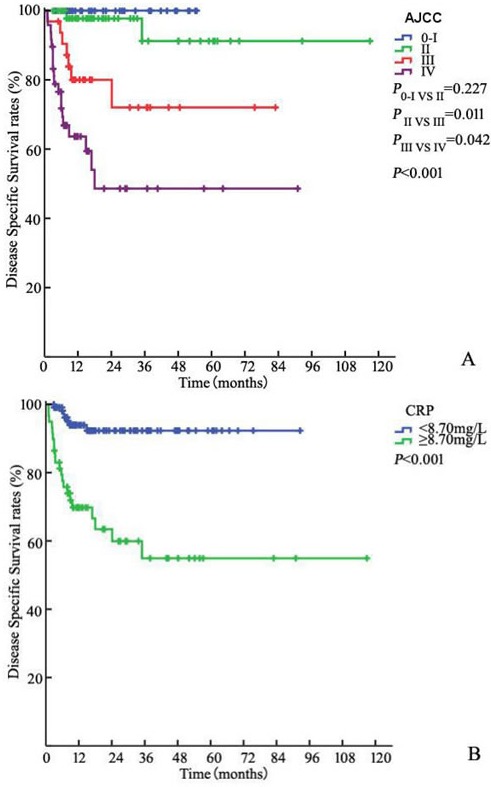

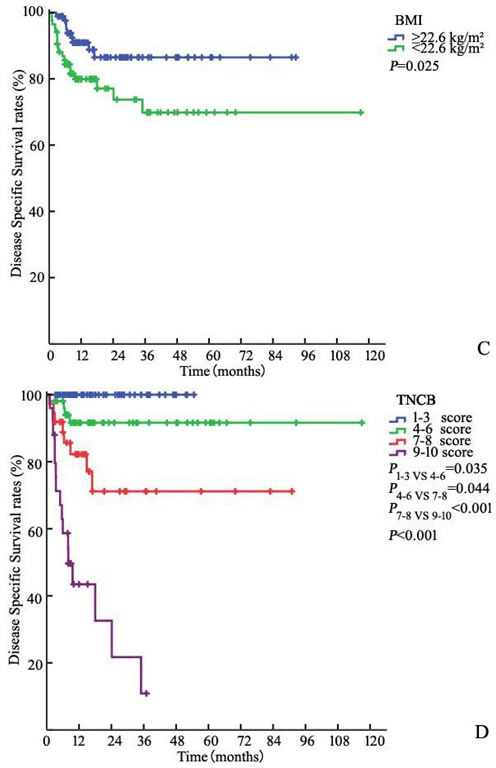
Kaplan-Meier estimates for DSS stratified by AJCC anatomic stage group **A.**, CRP **B.**, BMI **C.** and TNCB score group **D.**

Of all patients, 84 (48.8%) were LNM and 34 (19.8%) and 40 (23.3%) patients had bilateral LNM and ENE, respectively. Fifteen (17.8%) patients had stage pN1, 22 (26.2%) had stage pN2, and 47 (56.0%) had stage pN3. The 3-year DSS rates of patients with N1, N2, and N3 disease as determined by the 7th edition of the N staging system were 86.7%, 68.9% and 48.3%, respectively (*P* < 0.001). The corresponding DSS rates of patients at stages 0–I (39, 22.7%), II (53, 30.8%), III (32, 18.6%) and IV (48, 27.9%) disease according to the AJCC anatomic stage group were 100%, 91.2%, 72.0% and 48.2%, respectively (P0-I VS II = 0.227, PII VS III = 0.011, PIII VS IV = 0.042, *P* < 0.001, Figure 1A).

### CRP, BMI and survival

Of the 172 patients in the model development cohort, CRP increased preoperatively in 59 (34.3%). The 3-year DSS in patients with increased CRP (≥ 8.70 mg/L) was significantly worse than that in patients without increased CRP with rates of 54.9% and 92.3%, respectively (*P* < 0.001, Figure 1B). Similarly, there was a significant difference in DSS between patients with BMI < 22.6 kg/m² and BMI ≥ 22.6 kg/m² [86.5% and 69.9%, respectively (*P* = 0.025)] (Figure 1C).

### Survival predictor

Univariate analysis showed that BMI, pT stage, pN stage, grade, and preoperative CRP were associated with DSS (Table 1). Several risk factors for penile SCC, including smoking history and phimosis, which were identified by the guidelines, were added to the regression. Multivariate analysis revealed that BMI, pT stage, pN stage, grade, and preoperative CRP level were independent predictors of DSS (Table 2). A new scoring model was developed to predict DSS for penile cancer using the regression coefficients of the final multivariate model. The final model consisted of pT-stage, pN-stage, BMI and CRP (Table 2).

**Table 2:** Multivariate Cox regression analyses for DSS

Variable	Full model			Final model		
	HR	CI (95%)	*P*	HR	CI (95%)	*P*
BMI, kg/m^2^			0.002			0.002
< 22.6 VS ≥ 22.6	3.922	1.663-9.247		3.821	1.665-8.772	
CRP, mg/L,			0.006			0.004
≥ 8.70 VS < 8.70	3.849	1.472-10.065		3.706	1.531-8.972	
N stage			0.004			0.002
pN+ VS pN0	20.585	2.656-159.548		22.536	3.004-169.067	
**T**			< 0.001			< 0.001
≤ T1 VS T2 VS ≥ T3	5.504	2.520-12.020		5.023	2.371-10.641	
**G**			0.388	-	-	-
G1 VS G2 VS G3	1.307	0.712-2.401		-	-	-
Smoking History			0.520	-	-	-
No VS Yes	0.721	0.266-1.955		-	-	-
Phimosis			0.651	-	-	-
No VS Yes	1.280	0.440-3.724		-	-	-

HR = hazard ratio, CI (95%) = 95% confidence interval, Ref = reference.

### New scoring model

The score was calculated as 4 (if N+) + 3 (if ≥ pT3, T2 = 2, ≤ T1 = 1) + 2 (if BMI I < 22.6 kg/m²) + 1 (if CRP > 8.70 mg/dL) or 0 (if otherwise) (Table 3). The median score in the model development cohort was 5 (range 1 to 10, mean 5.1). The 3-year DSS values in patients with a score in the ranges 1–3 (low-risk, 58, 33.7%), 4–6 (intermediate-risk, 52, 30.2%), 7–8 (high-risk, 37, 21.5%) and 9–10 (very high-risk, 25, 14.5%) were 100%, 91.6%, 71.2% and 10.9%, respectively (P1-3 VS 4-6 = 0.035, P4-6 VS 7-8 = 0.044, P7-8 VS 9-10 < 0.001, *P* < 0.001 Figure 1D).

**Table 3:** TNBC model scoring

Variable(category)	Score
**N stage**	
pN+	4
pN0	0
**T**	
≤ T1	3
T2	2
T3	1
**BMI, kg/m$**,	
< 22.6	2
≥ 22.6	0
**CRP, mg/L**	
≥ 8.7	1
< 8.7	0

### Concordance index

In our study, we found that the addition of the pathological information significantly increased the predictive accuracy of the basic model (AJCC) (Table 4). We further evaluated discrimination and calibration. The C-index of the scoring model in patients in the model development cohort was 0.870. The bootstrap-corrected C-index of New Scoring Model was 0.871, which was inferior to that of the AJCC anatomic stage group (*P* < 0.001). The C-index of independent cohort were 0.820 and 0.867 for the AJCC anatomic stage group and TNCB score group.

**Table 4:** Predictive accuracy of models including the AJCC 7^th^ anatomic stage group system and the TNCB score group system

Group	P*	LR*	AIC	C-index	Bootstrap C-index （500 times）
**AJCC anatomic stage group**	0.001	35.745	121.080	0.817	0.818
**TNCB score group**	< 0.001	51.538	105.290	0.870	0.871

* Logistic, AIC = Akaike information criterion, LR = likelihood ratio, C-index = concordance index

## DISCUSSION

The preoperative levels of C-reactive protein (CRP) and body mass index (BMI) were determined and used to predict survival in patients with penile squamous cell carcinoma using data from a few studies. In the current study, we similarly investigated the prognostic value of molecular marker and physical status in patients with penile SCC. We firstly developed a new prediction scoring model including the pTN classification, CRP and BMI to predict disease specific survival. We term this algorithm the TNBC model.

Serum CRP is an indicator of acute or chronic inflammation [20]. Elevated CRP concentrations have been correlated independently with tumor load and disease progression, which have also been shown to correlate with survival in various cancers [10-12]. In our study, a significant correlation was shown between the CRP level and DSS in univariate analysis (*P* < 0.001, Figure 1B). Our results are consistent with those of several previous studies [13, 14]. Steffens, et al. also determined a correlation between high levels of CRP and progressive tumor characteristics, including ENE (*P* < 0.001), pelvic LNM (*P* = 0.007), pathologic tumor status (*P* = 0.002), and pathologic nodal status (*P* < 0.001). These pathological parameters are of the utmost prognostic importance in penile carcinoma [1, 6, 21]. Therefore, it is possible that elevated CRP would predict more aggressive and progressive disease. We presume that the addition of important information related to the status and extent of inguinal LNMs could increase the capacity for predictive migration and have a positive predictive effect on survival for micro-metastasis or tumor load.

Based on our findings, lower BMI may confer an advantage or may worsen prognosis. Although our results contradict those reported in Djajadiningrat's study [19], our results are very similar to many large-scale epidemiological studies that have revealed a significant correlation between BMI and a wide range of disease outcomes [16, 22, 23]. A recent study of 2,119 clear-cell renal cell carcinoma patients reported that patients with higher BMI had a better prognosis than their healthy-weight counterparts [24]. Based on an analysis using a Japanese multicenter database, lower BMI is associated with a worse prognosis in an Asian cohort with upper urinary tract urothelial carcinoma [25]. The first reason for this result is that the patients with aggressive and high-stage diseases are often lean, and there might be a reverse causation between lower BMI and risk of death from cancer [25]. The second reason may be that obese patients had lower expression levels of metabolism- and fatty acid-related genes than healthy-weight patients. Some of these genes were previously found to be overexpressed in tumors, conferring a growth advantage [15].

As far as we know, the present study is the first study to demonstrate the roles of CRP and BMI in penile SCC. In a multivariate analysis, elevated CRP and lower BMI were also found to be independent risk factors of DSS with TN classification. Therefore, based on regression estimates of significant parameters in multivariate analysis, we developed a new and simple outcome prediction model, which accounts for only four factors for penile SCC [26-28]. The TNCB score group system, which includes more of the above-mentioned parameters, significantly increased the predictive accuracy of the system of the AJCC. The new system also provides validated prognostic value by predicting DSS for patients with penile cancer (Table 4).

Recently, several prediction models have been presented for penile SCC based on small numbers [7-9]. However, they are difficult to implement in clinical practice because several pathological parameters are merely suggested, and physicians are uncomfortable determining which factors to include. They also use complex statistics. Unlike many of the other prognostic markers, BMI and CRP levels can be routinely tested in the clinic. In our present study, a molecular marker and physical status are combined to investigate the prognosis of penile SCC patients with comprehensive, simple and convenient methods. Our new model advances precision medicine and may facilitate multimodal treatment in patients.

We acknowledge that our study has some limitations. First, the data collection was retrospective in design. However, our study included the largest sample sizes with few changes in treatment paradigms. Second, adjuvant therapy and pelvic lymphadenectomy may potentially affect other parameters. Pelvic lymphadenectomy was not routinely performed before 2009 because the unified standard was not recommended in the guidelines. The adjuvant therapies were in varied forms, and the course of treatment was not unified. The results may still be subjected to selection bias that is inherent in this study design. Third, the limited number of patients and consequent events in this study also inhibited our ability to perform the optimal cutoff for these predictors and multivariate analyses. The the patient is N0 vs N+ with wider CIs in the HR than BMI/CRP for DSS. The inclusion of ≤T1 as one group which imparts an inherent selection bias and the authors may want consider subgrouping. However, we believe that this type of analysis will be important in future validation studies in larger multicenter data sets. Finally, the predictive accuracy of model should be externally validated in an independent population to determine its validity for clinical prediction. Thus, all analyses may be considered exploratory rather than hypothesis-tested. However, we believe that the present analysis will be important in future validation studies of larger and multicenter data sets.

Notwithstanding the above limitations, the TNCB score group system may increase the accuracy of survival prediction and aid in multimodal treatment planning for Chinese patients with penile cancer.

## PATIENTS AND METHODS

### Patient selection

From November 2005 to November 2014, we retrospectively reviewed the charts of 172 consecutive patients diagnosed with primary penile SCC at our institution. Serum CRP was measured 1–3 days before medical intervention cystectomy by immunoturbidimetry. Patients meeting the following criteria were excluded from the study: prior neo-adjuvant chemotherapy, previous surgery or radiotherapy of the inguinal region, clinical evidence of distant metastasis, and loss to follow-up. Based on the guidelines of the European Association of Urology (EAU), the treatment protocols were discussed with each patient. Lesions and lymph nodes (LNs) were pathologically confirmed. All histopathology reports were based on the 2014 American Joint Committee on Cancer (AJCC) TNM system. All patients provided written consent for storage of their information in the hospital database and for use of this information in our research. The Institutional Ethical Board of our hospital approved this study.

### Follow up

The deadline for follow-up was Oct. 2015. The follow-up period for each patient began at the time of cancer diagnosis and ended at death or the deadline. All patients were followed-up every 3 months for the first 2 years after surgery, every 6 months in the 3rd and 4th years, and then yearly thereafter.

### Statistical analyses

The optimal CRP cut off value to predict prognosis was calculated using a receiver operating characteristics (ROC) analysis referring to cancer-specific death [29]. Kaplan–Meier plots were used to estimate DSS using a log-rank test. We did not evaluate the role of adjuvant therapies using multivariate analyses because such therapies were not routinely administered to all of the enrolled patients. Several risk factors for penile SCC, which were identified by the guidelines, were added to the regression. Given that LNM laterality, extranodal extension (ENE) and pelvic LNM is included in the 7th N staging system, only additional factors were added to the regression. Multivariable Cox regression models were fitted to test the predictors of DSS. A new outcome prediction model for DSS was developed based on the regression coefficients from the final multivariate Cox proportional hazards model [26-28]. The likelihood ratio (LR), Akaike information criterion (AIC), and the concordance index (C-index) were investigated to evaluate the accuracy of the models. Bootstrap corrected c-indexes were used to internal validation for better gauge expected future predictive accuracy (500 times sampling). One separate, independent dataset including 74 patients from 2 institutions (Sun Yat-sen University Cancer Center(23 patient, December 2014-March 2015), Guangzhou Cancer Center of Guangzhou Medical University (51 patient, March 2004-November 2014) were used to external validation. All of the statistical analyses were performed with R2.11.1 http://www.r-project. org), and a two-sided P < 0.05 indicated significance.
